# Supplementary material for: Giant dynamic electromechanical response via field driven pseudo-ergodicity in nonergodic relaxors
Source: Nat Commun. 2023 Apr 27;14:2414. doi: 10.1038/s41467-023-38006-6 (PMC10140180; doi:10.1038/s41467-023-38006-6)
Supplement: Supplementary file 1 — Supplementary Information [file 41467_2023_38006_MOESM1_ESM.pdf]

Supporting Information

Giant dynamic electromechanical response via field driven pseudo-ergodicity in nonergodic relaxors

He Qi [1,2], Tengfei Hu[3], Shiqing Deng[2], Hui Liu[2], Zhengqian Fu[3] ✉, Jun Chen[1,4] ✉

[1]Beijing Advanced Innovation Center for Materials Genome Engineering, Department of Physical Chemistry, University of Science and Technology Beijing, Beijing 100083, P. R. China
[2]School of Mathematics and Physics, University of Science and Technology Beijing, Beijing 100083, P. R. China
[3]State Key Laboratory of High Performance Ceramics and Superfine Microstructures, Shanghai Institute of Ceramics, Chinese Academy of Sciences, Shanghai 200050, P. R. China
[4]Hainan University, Haikou 570228, Hainan Province, China

E-mail: junchen@ustb.edu.cn, fmail600@mail.sic.ac.cn

H. Qi and T.F. Hu contributed equally to this work.

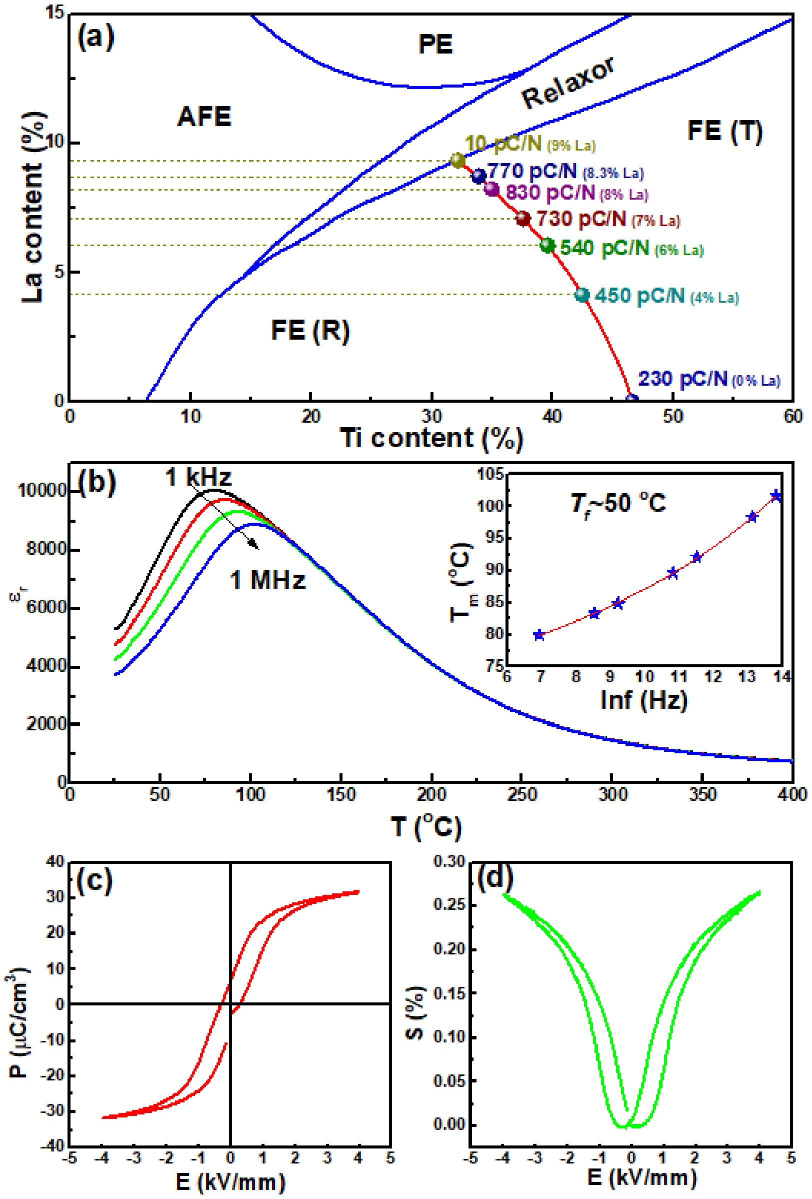

Fig. S1 Electrical properties of (Pb,La)(Zr,Ti)O_3 system. (a) Phase diagram and piezoelectric properties of (Pb,La)(Zr,Ti)O_3 system. (b) Temperature and frequency dependent dielectric permittivity of $PL_9Z_{67}T_{33}$ ceramic. According to the *V-F* fitting result shown in the inset, the freezing temperature of $PL_9Z_{67}T_{33}$ ceramic is about T_f~50 °C. Namely, it shows a nonergodic relaxor state at room temperature. Room-temperature (c) *P-E* and corresponding (d) *S-E* curves of $PL_9Z_{67}T_{33}$ ceramic. Pinched *P-E* loop and sprout shape S-E curve without negative strain can be seen for this sample, showing obvious reversible transition under electric field.

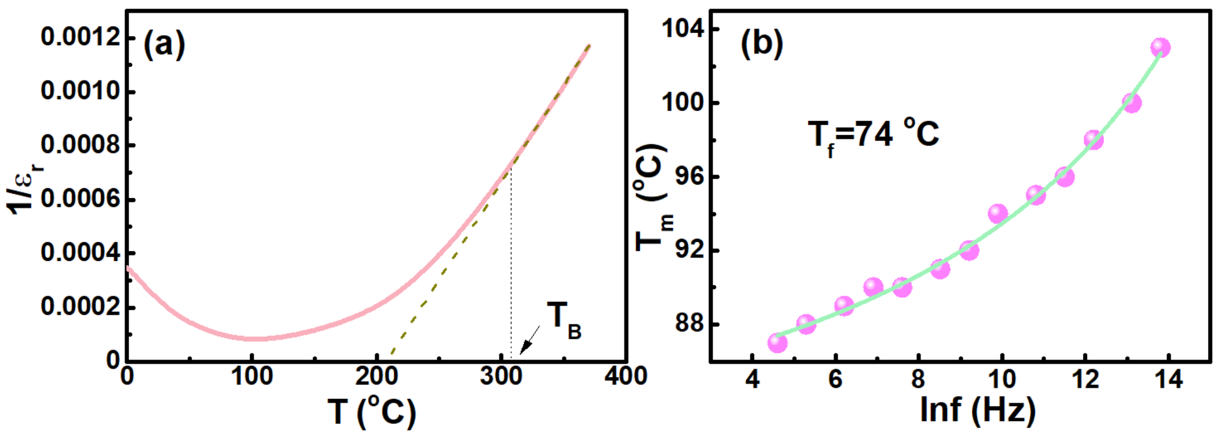

Fig. S2 The calculation of critical temperatures for ergodic relaxor phase zone. (a) The deviation from the *Curie-Weiss* law and (b) *Vogel-Fulcher* fitting of the temperature of the maximum dielectric permittivity as a function of measurement frequency.

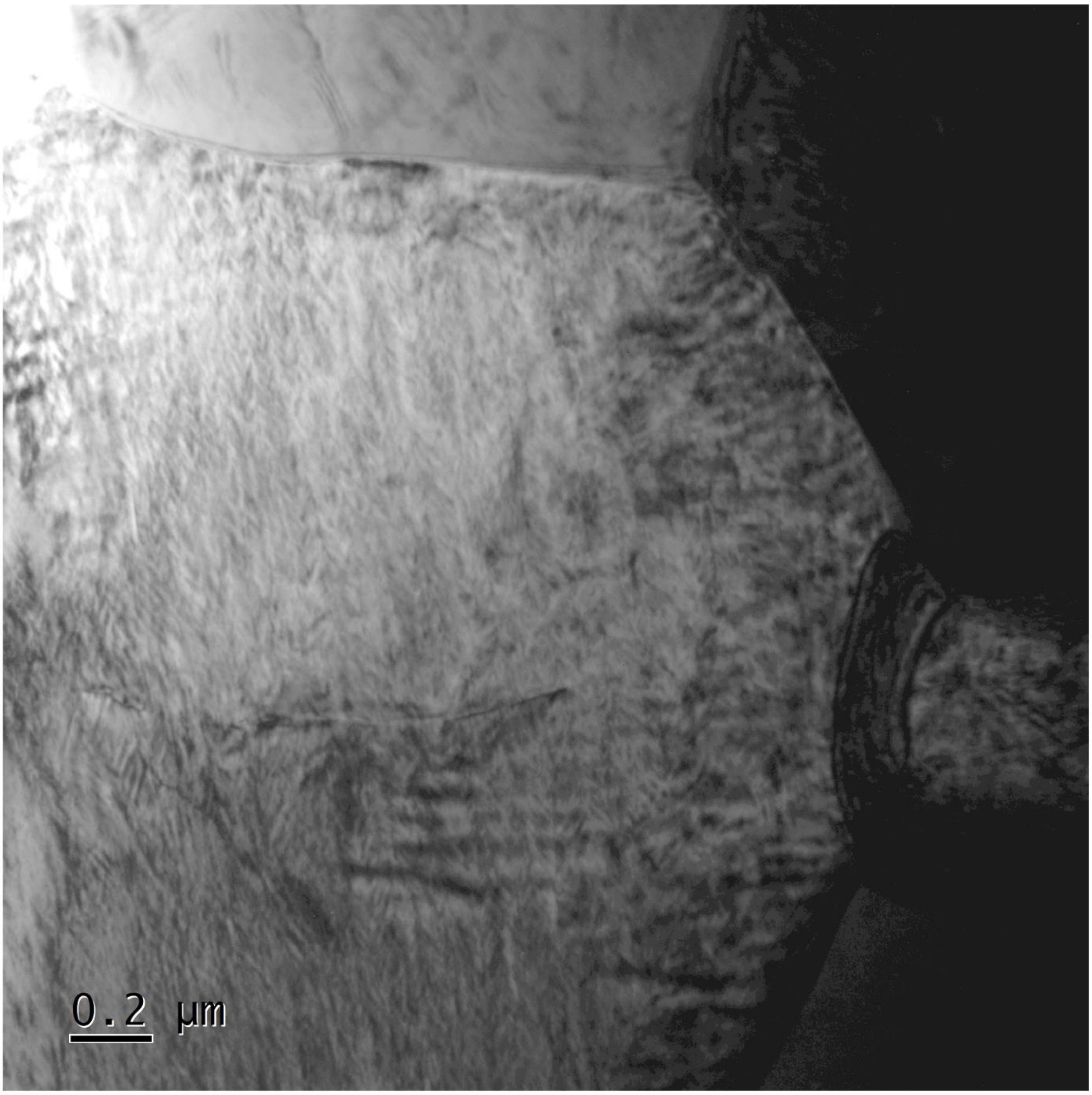

Fig. S3 Bright field TEM image along <100>$_c$ direction for $PL_{8.3}Z_{66}T_{34}$ ceramic.

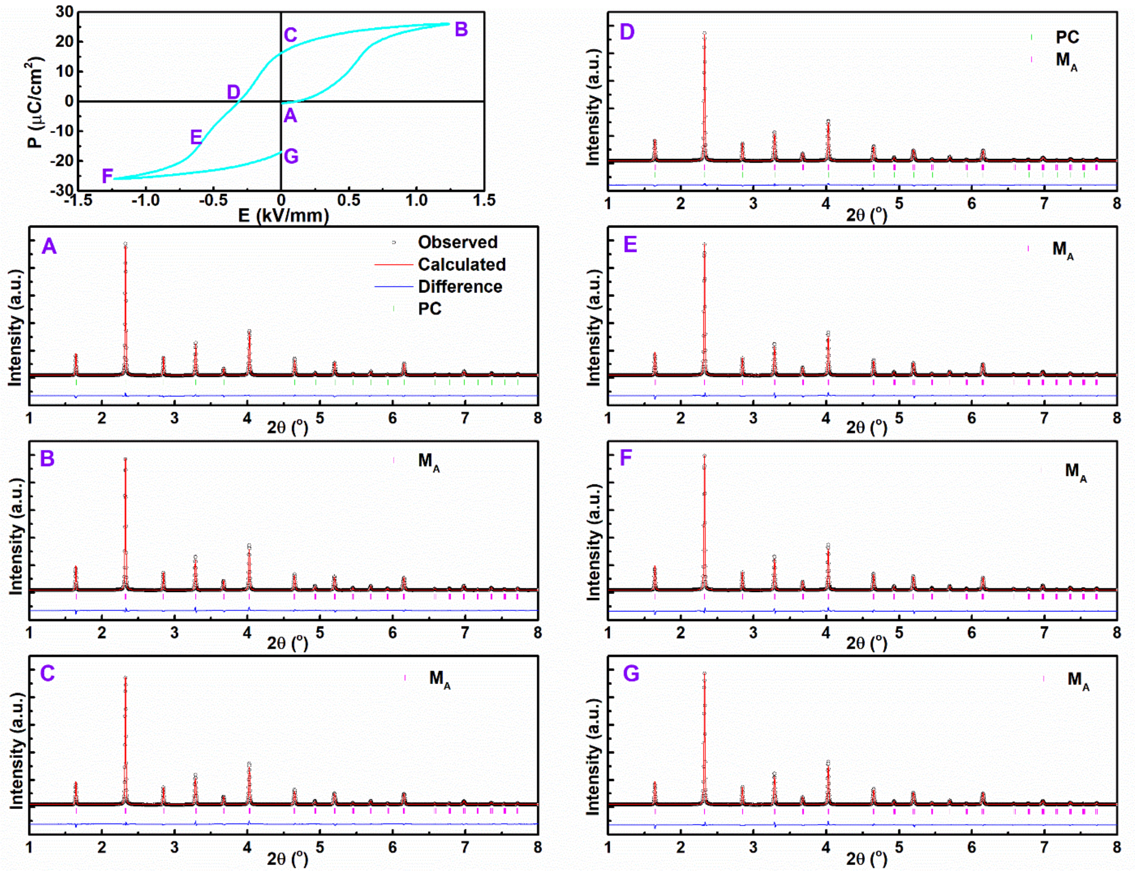

Fig. S4 Phase structure evolution under electric field. Rietveld refinement for the SXRD measured under different electric field at ϕ=45° for $PL_{8.3}Z_{66}T_{34}$ ceramic. The corresponding electric fields are marked on the *P*-*E* loop.

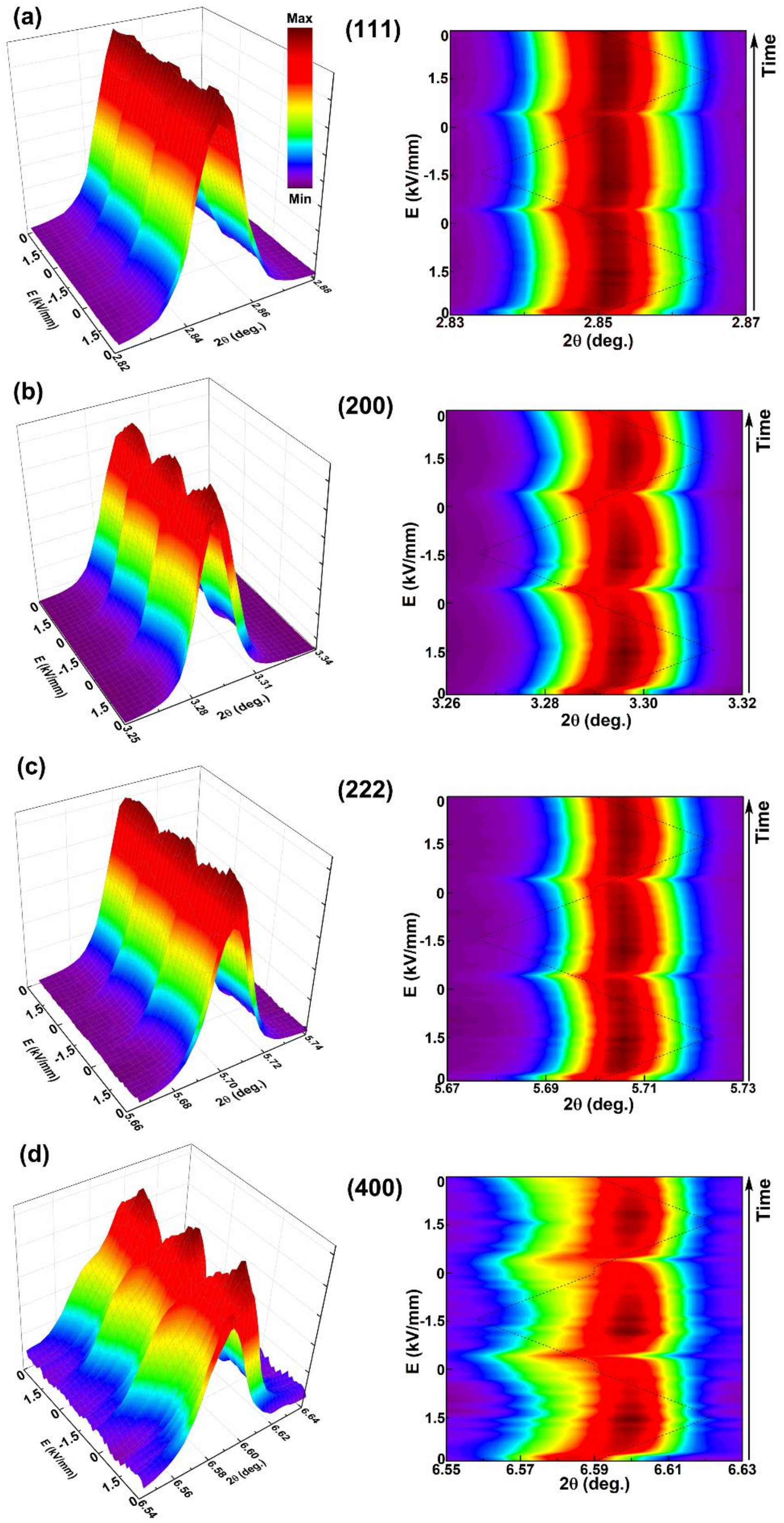

Fig. S5 Evolution of several diffraction peaks as function of bipolar electric field at ϕ=90º: (a) *(111)*, (b) *(200)*, (c) *(222)* and (d) *(400)* for $PL_{8.3}Z_{66}T_{34}$ ceramic.

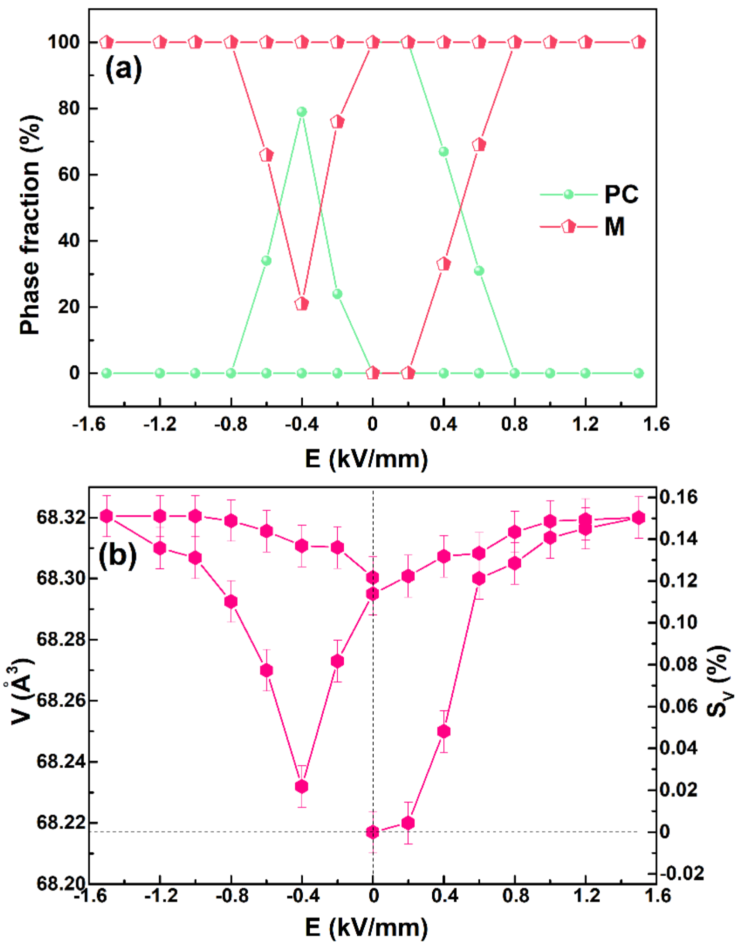

Fig. S6 Phase structure parameter evolution under electric field. Quantification of (a) phase fraction and (b) unit cell volume (normalization using basic perovskite unit cell) and volumetric change under different electric fields. The data are presented as the mean ± SEM.

The volumetric change between the initial (V_0) and resultant phases (V_i under E=i kV/mm) can be calculated by the equation:

$$S_V = (V_i - V_0)/V_0 - 1$$

Thus the volumetric strain in each respective direction of the sample that results from the volumetric change can be given by:[Acta Mater., 58, 2103-2111 (2010)]

$$S_{V,33} = S_V / 3$$

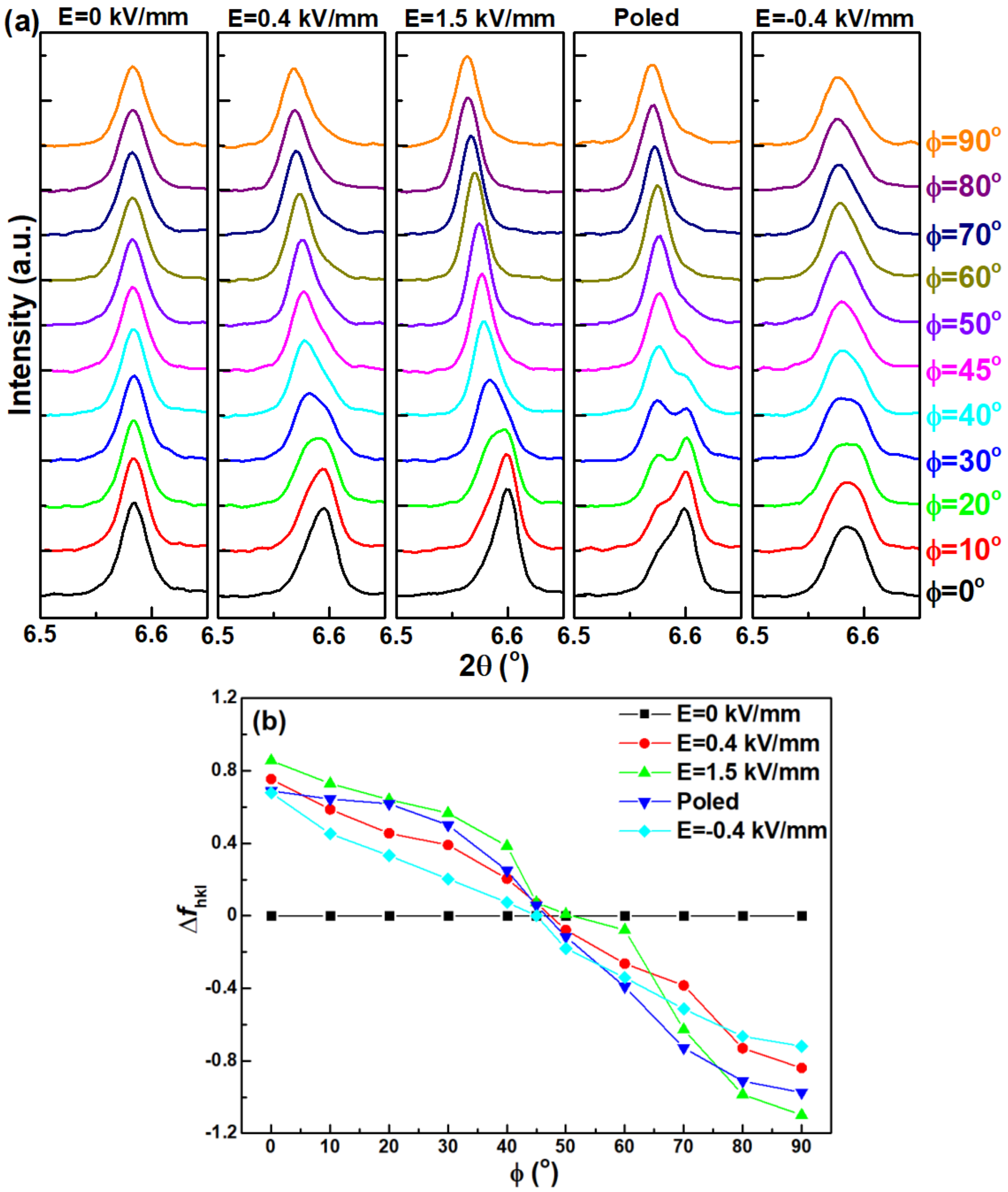

Fig. S7 The domain texture information under different electric fields. (a) Orientation dependent $(400)_c$ diffraction peaks under different electric fields. (b) Δf_{hkl} calculated from $(400)_c$ diffraction peaks under different electric fields at different azimuthal angles ϕ.

The change in multiples of a random distribution of monoclinic phase (*Cm* space group) can be calculated according to the diffraction intensity change between $E=i$ kV/mm and a randomly distributed state ($I_{0,440M}$:$I_{0,004M}$=2) using the following equation:

$$\Delta f_{hkl} = 3\cdot\frac{I_{i,440M}/I_{0,440M}}{[I_{I,440M}/I_{0,440M}+(I_{I,004M}/I_{0,004M})/2}-2$$

Considering the PC phase as a randomly-textured M phase with a small lattice distortion, thus the macroscopic strain due to the texture of M phase to the electric field direction within a polycrystalline sample can be calculated by[J. Am. Ceram. Soc., 92, 2300 (2009)]

$$S_{text,33} = \frac{a(\alpha) - c(\alpha)}{c(\alpha)} \int_{\alpha=0}^{\pi/2} [\Delta f_{hkl}(\alpha) \times \cos^2 \alpha](\sin \alpha) d\alpha$$

Where α is the sample direction with respect to the applied electric field (α=0°). The geometrical factor of $cos^2\alpha$ in the integrand arises from a tensorial transformation of the domain wall motion strain along the direction of the applied electric field. The factor $sin\alpha$ describes the transformation from an elemental volume in the orientation space to an effective volume fraction within the sample.[J. Appl. Phys., 97, 034113 (2005), J. Appl. Phys., 98, 024115 (2005)].

The electric field induced lattice strains of each crystal plane can be calculated following:

$$s_{hkl} = (d_{i,hkl} - d_{0,hkl}) / d_{0,hkl}$$

where $d_{i,hkl}$ and $d_{0,hkl}$ are (*hkl*) lattice spacings of E=*i* kV/mm and E=0 kV/mm, respectively. The total lattice strain S_L can be given as:

$$S_L \cong \sum_{hkl} T_{hkl} m_{hkl} s_{hkl} / \sum_{hkl} T_{hkl} m_{hkl}$$

where m_{hkl} and T_{hkl} represent the planar multiplicity and the texture factor (multiples of a random distribution) of the respective domain orientations parallel to the electric field after poling of this material.[J. Appl. Phys. 101, 094104 (2007)] Here, the lattice strain is calculated according to the shift of $(440)_M$ and $(004)_M$ peaks at ϕ=0° under different electric fields.

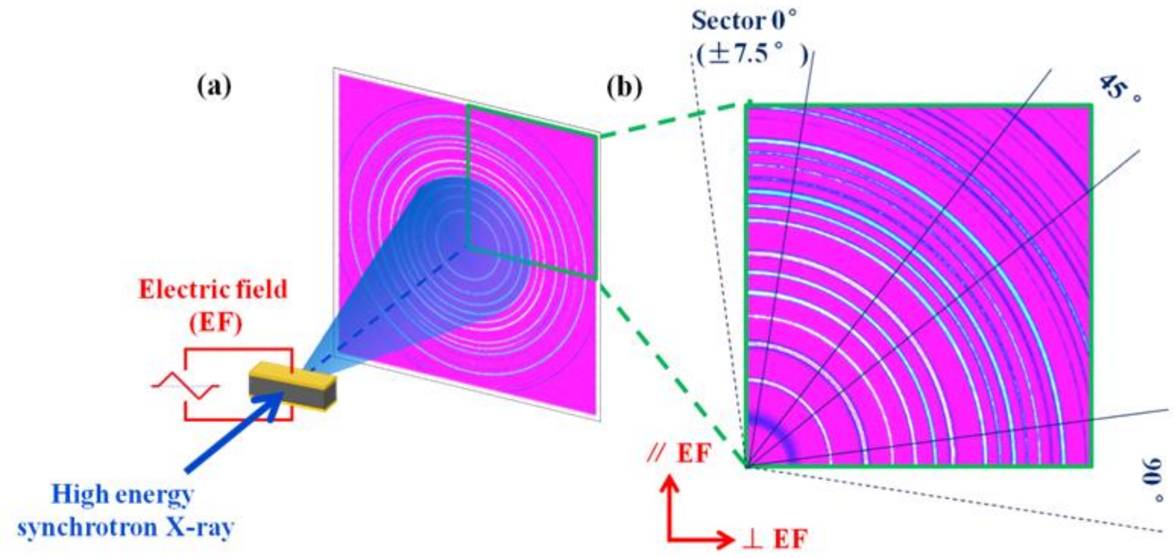

Fig. S8 Schematic of the experimental setup for in-situ high-energy synchrotron X-ray diffraction experiments.

For the in-situ high-energy SXRD measurements, the ceramic samples were polished, cut into rectangular bars with dimensions of 6 mm×1 mm×0.6 mm, and then annealed at 500 °C for 6 h to release stresses. Gold electrodes with a size of 6 mm×1 mm were evaporated on opposite faces of the bars. The in-situ high-energy SXRD measurements were conducted at the 11-ID-C beamline at the Advanced Photon Source (APS) at the Argonne National Laboratory. The X-ray beam with a spot size of 0.5 mm×0.5 mm, photon energy of about 110 keV and a wavelength of 0.11165 Å were used. A Perkin Elmer amorphous silicon 2D detector, positioned approximately 1800 mm away from the sample, was used to collect the scattered photons. The direction of the electric field was perpendicular to the X-ray beam. A triangular bipolar cycle electric field with a step of about 0.15-0.2 kV mm^{-1} was applied to the ceramic samples. At each step, a diffraction pattern was recorded with an acquisition time of 30 s, which is equivalent to a cyclic frequency around 40 mHz. The diffraction pattern of Ceria standard was used to calibrate the detector related parameters. The Debye rings collected on the 2D detector were divided into equidistant sectors at 15°. Intensities of the X-ray diffraction peaks in the sectors were integrated using the Fit2d software.

Table S1: Lattice parameters and refined structure parameters of $PL_{8.3}Z_{66}T_{34}$ ceramic under different electric field conditions according to Rietveld refinement results shown in Fig. S4.

**Condition**	**A**	**B**	**C**	**D**		**E**
**E (kV/mm)**	**0**	**1.4**	**0**	**-0.4**		**-0.6**
Space group	$Pm\bar{3}m$	*Cm*	*Cm*	$Pm\bar{3}m$ (79%)	*Cm* (21%)	*Cm*
a (Å)	4.0860(1)	5.7909(1)	5.7906(1)	4.0863(1)	5.7898(1)	5.7906(1)
b (Å)	4.0860(1)	5.7812(1)	5.7810(1)	4.0863(1)	5.7798(1)	5.7805(1)
c (Å)	4.0860(1)	4.0814(1)	4.0802(1)	4.0863(1)	4.0779(1)	4.0805(1)
α (°)	90	90.101(3)	90.091(2)	90	90.078(2)	90.098(3)
Cell volume (Å^3)	68.217(2)	136.640(3)	136.590(3)	68.232(2)	136.463(3)	136.585(3)
R_w (%)	2.45	2.27	2.32	2.40		2.43
GOF	1.96	1.84	1.87	1.92		1.95

**Condition**	**Atoms**	***x***	***y***	***z***	**fraction**	**Uiso**
**A** $Pm\bar{3}m$	Pb	0	0	0	0.917	0.03607
	La	0	0	0	0.083	0.04469
	Ti	0.5	0.5	0.5	0.35	0.01160
	Zr	0.5	0.5	0.5	0.65	0.01160
	O	0.5	0.5	0	1	0.05260
**B** ***Cm***	Pb	0.0005	0	-0.0012	0.917	0.03688
	La	0.0005	0	-0.0012	0.083	0.03688
	Ti	0.4717	0	0.5328	0.35	0.01153
	Zr	0.4717	0	0.5328	0.65	0.01153
	O1	0.5006	0	0.1226	1	0.07280
	O2	0.2195	0.2588	0.5936	1	0.07280
**C** ***Cm***	Pb	0.0002	0	-0.0002	0.917	0.03875
	La	0.0002	0	-0.0002	0.083	0.03875
	Ti	0.4689	0	0.5209	0.35	0.00413
	Zr	0.4689	0	0.5209	0.65	0.00413
	O1	0.433	0	0.0918	1	0.05656
	O2	0.2282	0.2798	0.5774	1	0.05656
**D** $Pm\bar{3}m$	Pb	0	0	0	0.917	0.03364
	La	0	0	0	0.083	0.03364
	Ti	0.5	0.5	0.5	0.35	0.03797
	Zr	0.5	0.5	0.5	0.65	0.03797
	O	0.5	0.5	0	1	0.07083
**D** ***Cm***	Pb	0	0	0	0.917	0.03271
	La	0	0	0	0.083	0.03271
	Ti	0.4703	0	0.5204	0.35	0.02426
	Zr	0.4703	0	0.5204	0.65	0.02426
	O1	0.4211	0	0.1016	1	0.02548
	O2	0.2270	0.2809	0.5763	1	0.02548
**E** ***Cm***	Pb	0.0003	0	-0.0004	0.917	0.03886
	La	0.0003	0	-0.0004	0.083	0.03886
	Ti	0.4726	0	0.5196	0.35	0.01050
	Zr	0.4726	0	0.5196	0.65	0.01050
	O1	0.4474	0	0.1109	1	0.08934
	O2	0.2305	0.2759	0.5795	1	0.08934
